# Local, Early, and Precise: Designing a Clinical Decision Support System for Child and Adolescent Mental Health Services

**DOI:** 10.3389/fpsyt.2020.564205

**Published:** 2020-12-15

**Authors:** Thomas Brox Røst, Carolyn Clausen, Øystein Nytrø, Roman Koposov, Bennett Leventhal, Odd Sverre Westbye, Victoria Bakken, Linda Helen Knudsen Flygel, Kaban Koochakpour, Norbert Skokauskas

**Affiliations:** ^1^Department of Computer Science, The Norwegian University of Science and Technology, Trondheim, Norway; ^2^Regional Centre for Child and Youth Mental Health and Child Welfare, Department of Mental Health, Faculty of Medicine and Health Sciences, Norwegian University of Science and Technology (NTNU), Trondheim, Norway; ^3^Regional Centre for Child and Youth Mental Health and Child Welfare (RKBU) Northern Norway, The Arctic University of Norway (UiT), Tromsø, Norway; ^4^Sechenov First Moscow State Medical University, Moscow, Russia; ^5^Department of Psychiatry, Division of Child and Adolescent Psychiatry, The University of California San Francisco, San Francisco, CA, United States; ^6^Department of Child and Adolescent Psychiatry, St. Olav's University Hospital, Trondheim, Norway; ^7^Department of Mental Health, Haukeland University Hospital, Bergen, Norway

**Keywords:** child and adolescent mental health, clinical decision support system (CDSS), clinical decision support (CDS), innovation & technology strategy, child and adolescent psychiatry (CAP), child and adolescent mental health services (CAMHS)

## Abstract

Mental health disorders often develop during childhood and adolescence, causing long term and debilitating impacts at individual and societal levels. Local, early, and precise assessment and evidence-based treatment are key to achieve positive mental health outcomes and to avoid long-term care. Technological advancements, such as computerized Clinical Decision Support Systems (CDSSs), can support practitioners in providing evidence-based care. While previous studies have found CDSS implementation helps to improve aspects of medical care, evidence is limited on its use for child and adolescent mental health care. This paper presents challenges and opportunities for adapting CDSS design and implementation to child and adolescent mental health services (CAMHS). To highlight the complexity of incorporating CDSSs within local CAMHS, we have structured the paper around four components to consider before designing and implementing the CDSS: supporting collaboration among multiple stakeholders involved in care; optimally using health data; accounting for comorbidities; and addressing the temporality of patient care. The proposed perspective is presented within the context of the child and adolescent mental health services in Norway and an ongoing Norwegian innovative research project, the Individualized Digital DEcision Assist System (IDDEAS), for child and adolescent mental health disorders. Attention deficit hyperactivity disorder (ADHD) among children and adolescents serves as the case example. The integration of IDDEAS in Norway intends to yield significantly improved outcomes for children and adolescents with enduring mental health disorders, and ultimately serve as an educational opportunity for future international approaches to such CDSS design and implementation.

## Introduction

Nearly one half of mental health problems develop prior to the age 15 (1) and 75% of all psychiatric disorders have their onset prior to the age of 25 (2–4). In Norway, one out of five children has a mental disorder at any point in time (5, 6) and nearly five percent of all children and adolescents receive treatment in child and adolescent mental health services (CAMHS) (7, 8).

Modern electronic health records (EHRs) provide detailed documentation of a patient's health, but the complexity of psychiatric and neurodevelopmental disorders in childhood and adolescence requires clinical decision-making support beyond the EHRs' scope (9, 10). EHRs rarely provide adequate insight into the complex situations of psychiatric care, including recently updated biological frameworks for disorders and emerging methods for identifying syndromes (11–13). The incorporation of telepsychiatry and other computer supported health approaches can efficiently utilize existing resources to improve evidence-based early intervention and preventative CAMHS (13–15).

### Clinical Decision Support Systems

A clinical decision support system (CDSS) aims to provide clinicians with real-time, step-by-step guidance through their clinical decision-making process (16–18). A CDSS intends to provide recommendations and guidance, not to replace the clinical judgment of practitioners. In general, a CDSS can be designed to rely solely on clinical practice guidelines to provide the evidence-based support, and/or incorporate previous patient cases by including healthcare datasets (18). The construction of guidelines for a CDSS is typically done with guideline development tools and computer-interpretable guideline (CIG) modeling languages, such as PROforma and SAGE (19). However, depending on the specific purpose of the CDSS, relying on modeled guidelines alone could be a suboptimal approach (20, 21). Traditional CDSS design and implementation aspects critical to successful CDSS adoption have included: (1) integration and adaptation to workflow; (2) construction of the information system structure and components; (3) knowledge management, interoperability, and sharing; (4) cognitive tasks and reasoning processes to be supported; (5) health system priorities and CDSS adoption paradigms; (6) quality improvement impacts, and (7) evaluation of effectiveness of decision support intervention (21, 22).

### Child and Adolescent Mental Health Services in Norway

Norway is one of many Western nations that use an integrated approach for CAMHS. A family member, a teacher, or school counselor usually serves as the initial contact for children experiencing mental health problems, and refers them to a care provider. For example, if a teacher notices a child is challenged academically, they will involve the Educational and Psychological Counseling Service (PPT), which assesses the problem and determines whether special education assistance is an appropriate intervention, or if involvement of different local, regional, or national services is most appropriate for the child (23).

Typically children are first referred to their local primary care provider (PCP) for further assessment. If the mental health problem is more complex in nature, a PCP needs to involve additional services from professionals who are trained to address such problems. For example, if there are child safety and well-being concerns, child protection services are involved, and if a child requires assessment and/or interventions by a child and adolescent psychiatrist, a referral to CAMHS is made (23, 24).

In addition to Norway's standardized, integral approach to patient assessment and treatment, the Norwegian Directorate of Health has also established national clinical guidelines and care pathways (i.e., Pakkeforløp in Norwegian) for several mental health disorders, similar to the United States' American Academy of Child and Adolescent Psychiatry (AACAP), formation of clinical updates and practice guidelines (25, 26). The national guidelines and standardized pathways help to improve the predictability and safety of care and facilitate collaboration between the different services involved (23, 27, 28).

## CDSS Design in the CAMHS Context

While CDSS implementation for general medicine has been well researched, the use of CDSS in CAMHS has been limited, with only a handful of studies focusing specifically on CAMHS, and many reporting shortcomings (11, 12, 18). CDSS design for CAMHS requires careful consideration of the complexity of the care process. The design and implementation should therefore take into consideration not only the previously documented challenges but also the structure and needs of local CAMHS (10–12).

To structure our discussion of the care context that a CAMHS CDSS must support, we have identified four key design considerations, representing (1) the collaborative aspect of mental health care, (2) the many and distributed sources of information, (3) the complexity introduced by multiple stakeholders and comorbidities, and (4) the long-term perspective of the care process.

### A CDSS for Collaborative Care

Traditionally, standardized clinical guidelines and care pathways are designed for healthcare professionals directly involved in clinical care. But, providing quality care needs to involve all stakeholders, including teachers, community mentors (i.e., youth groups), coaches, as well as the patients and their families. Similar to clinical guidelines, traditionally CDSSs focus on the clinical provider and assists one individual through clinical decision-making (i.e., a psychologist or PCP) (22). There are several practical reasons for this, including legacy EHR systems' minimal interoperability, yet such approaches limit the scope of CDSS functionality, especially in CAMHS.

To maximize the value, usefulness, and impact of a CDSS, the correct information must reach all relevant stakeholders, whether directly or indirectly engaged (29). As the patient is the most important stakeholder in his or her own care, their active participation helps them to better understand the treatment, and ultimately improves disease self-management (30, 31). In Norway, the Patients' Rights Act stipulates that all Norwegian citizens have the legal right to participate in their own care (32). Children and adolescents can provide consent and have a parent serve as a proxy (32, 33). Previous CDSS studies have shown CDSS system design should consider involvement of a parent as a proxy, as it increased patients' adherence to CDSS recommendations (17).

### A CDSS for Application of Health Data

In a typical clinical scenario, decision-making is based on the patient's EHR, data from an associated patient database, and single-user data entry. The EHR should provide a holistic, comprehensive overview of the patient's health to maintain a consensus among all stakeholders involved in the patient's care. Assessment tools help identify the extent of a patient's problems and which stakeholders to involve in the patient's care. Self-reporting of symptoms has also become more common with the increased use and popularity of digital and web-based tools, especially among children and adolescents (34, 35). These methods of collecting information from multiple stakeholders involved, contributes to establishing a clearer picture of a patient's health. Clear communication and efficient sharing of the patient's health information is needed to provide the best quality care for each patient, as challenges with poor information flow and transparency directly affect the quality of care (36). A collaborative CDSS design, where multiple stakeholders participate in data collection and data entry, would increase the CDSS's utility as well as improve information flow among stakeholders (10).

Design of CDSS guidance based on analysis of health datasets has been found to provide greater improvement of clinical decision-making than guideline based CDSS suggestions alone (37). The data-driven approach to CDSS design can, not only provide decision-making support beyond the capacity of clinical guidelines, but also provide clinical learning opportunities (38). Reported secondary benefits of data-driven CDSS have included enhancing education, expanding research knowledge, improving guideline adherence, and clarifying training needs (39). Extending the role of a CDSS in this way can yield positive outcomes for patients with the most complex psychiatric needs.

### A CDSS to Address Stakeholder Perspectives & Comorbidities

Applying a CDSS in clinical CAMHS also faces a challenge related to “cognitive collaboration” (40). “Cognitive collaboration” involves distributed cognitive processes from all stakeholders contributing to care, whose expertise covers a variety of professions (40, 41). Despite their common goal of helping the patient, the stakeholders' criteria for success, and their approaches to achieve that goal, may differ. For example, a school counselor's perspective on aspects of the clinical process may differ from that of a psychiatrist. A CDSS designed for one aspect of treatment might optimally address that particular focus, but this design approach could be less relevant to the overall clinical profess if it neglects the “cognitive collaboration” involved in care (42).

In addition to multiple cognitive perspectives, the CDSS design also needs to account for comorbidities. Approximately 40% of all children and adolescents who meet the criteria for one disorder (i.e., anxiety, behavior, mood, or substance-use disorders) also meet the criteria for another disorder (43). Without considering abnormal symptomatic display or symptom overlap, comorbidity patterns can be concealed and mislead the practitioner to provide an invalid diagnosis (44). However, most CDSS models do not account for comorbidities, and research is scare on how to apply multiple CIGs, in order to do so (11, 12, 45). A CDSS for CAMHS needs to be able to account for commonly occurring comorbidities, as well as the collaborative nature of clinical care (46).

### A CDSS for Temporality of Care

In Norway, the Patients' Rights Act guarantees every individual the right to immediate, appropriate care (32). For example, if a patient is referred to a psychologist or psychiatrist, they have the right to be seen within ten working days, and even sooner if the illness is deemed life-threatening (23, 32). Despite such policies, the patient's care progress and overall improvement of health can be delayed. Misdiagnosis, for example, can arise with child and adolescent mental health disorders due to the large variations in frequency, severity, and types of symptoms displayed, such as with ADHD (10).

Reaching a clinical diagnosis is only the first step in a complex and collaborative care process. A 2009 study on medical treatment for children with ADHD, found that only about half of the cohort managed to adhere to the ADHD medication plan (47). For a CDSS to be relevant to all components of the care process, potential complications that could arise in treatment management and follow-up also need to be taken into account. For example, a CDSS could be designed to consider any developments between appointments, or to register any irregularities prescribed medications and automatically alert the practitioner (48). To date, CDSS implementation and evaluations have predominantly focused on short-term outcomes rather than long-term care for the patient (42). While CDSS design has not yet optimally addressed the longitudinal and collaborative nature of patient care, many CIG modeling languages that can be used for a CDSS (i.e., EON, GASTON, etc.) do (49). In addition to utilizing a CIG language with longitudinal context, it is essential to assess how the different components of temporality of clinical care, and the specific timing of each intervention step, can impact the use of a CDSS (42).

## A Complex Proposition to Meet Complex Needs: The IDDEAS Project

The complexities of a CDSS for CAMHS have all come under consideration in the development of the Individualized Digital DEcision Assist System (IDDEAS) project. IDDEAS, an innovation and research project in Norway, aims to design and implement a CDSS that can support the diagnoses and treatment of mental health disorders in children and adolescents, starting with ADHD as the first model clinical paradigm (50, 51). With nearly 4% of all 12 year olds in Norway having ADHD at any point in time (7), the disorder will serve as the first case example for IDDEAS. IDDEAS brings innovation to patient care to allow earlier and more precise clinical decision-making.

The main goal of IDDEAS is to develop a CDSS that will improve mental health outcomes for children and adolescents by supporting the practitioner through clinical decision-making. IDDEAS specifically seeks to improve care by providing clinicians data-driven and evidence-based guidance in real time, to ensure earlier and more precise decision-making, avoid misdiagnosis and inefficient care practices, and improve individualized treatment management. In addition to the Norwegian CAMHS guidelines and clinical care pathways, IDDEAS will also use Norway's unique and existing resources—CAMHS datasets and other health datasets—to provide data-driven support.

**Figure 1:**
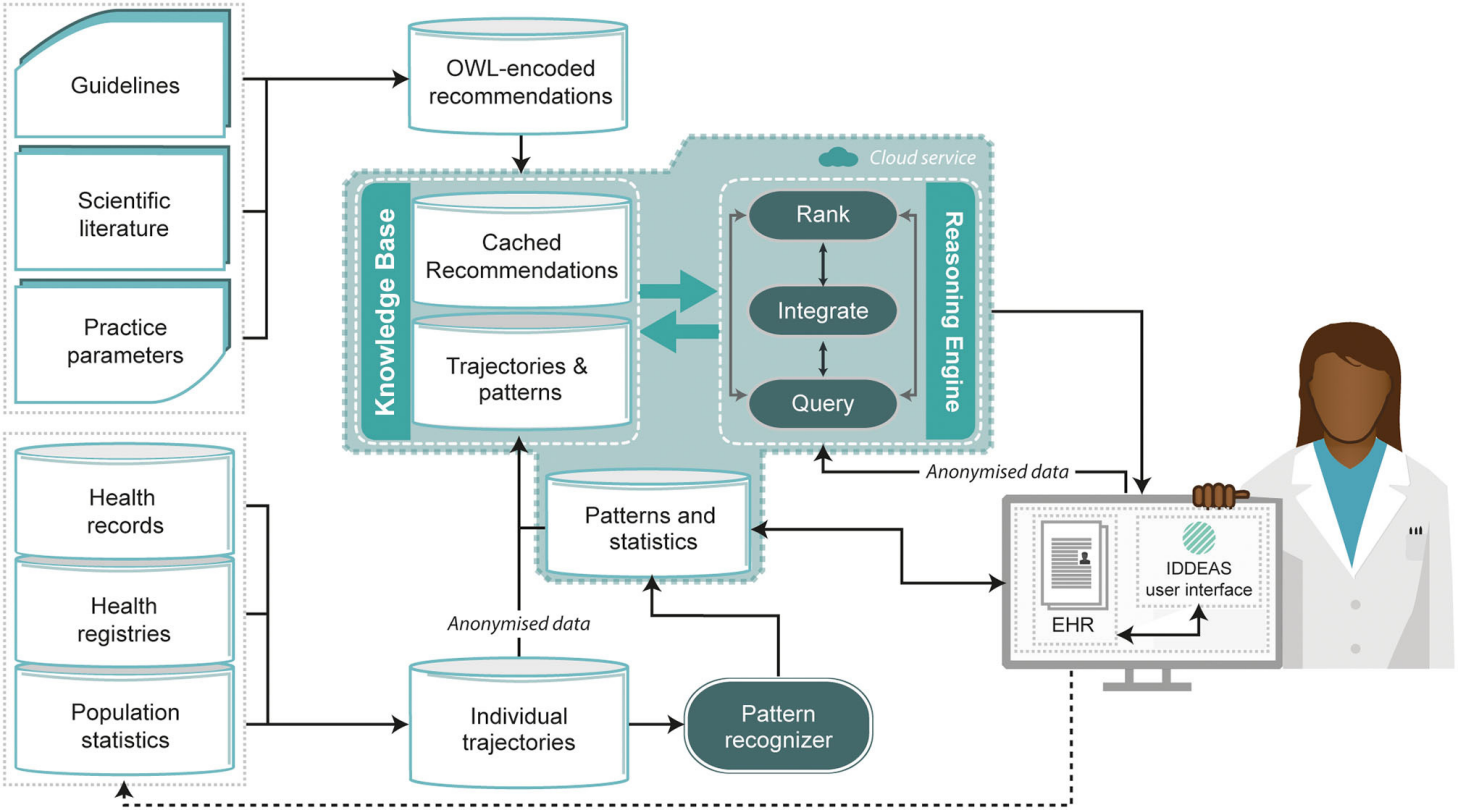
The IDDEAS clinical decision support system model. This figure is adapted from (50) which was published under the CC BY 4.0 license (https://creativecommons.org/licenses/by/4.0/). It has been slightly revised to reflect architecture development.

The central and most important innovation in IDDEAS is the *L*ocal *E*arly and *P*recise (LEaP) model, which allows for the application of IDDEAS *locally* in community settings, *early* in the clinical process, to add *precision* to patient care. The LEaP model is designed to provide real-time decision support for busy practitioners. IDDEAS integrates existing heterogeneous, geographically distinct, current and historical datasets, to generate new information and models to provide clinical decision support at the individual patient level (Figure 1). Data representing multiple episodes of care for different patients are structured into domains of inter-related concepts and hierarchical clinical patterns. They are then ranked within the system, matched with the current patient and ultimately provided within the system's interface to support the practitioner through clinical decision making (50, 51). In addition, guidelines and other clinical recommendations are compiled and encoded before being combined with the data-driven trajectories and patterns to provide ranked suggestions in response to any practitioner queries (50, 51). By designing a CDSS that utilizes both guidelines and big data, the system has the potential to be curated based on evolving scientific evidence, and with the use of each individual patient's own EHR data to also build upon the available evidence base within the system (51).

The IDDEAS CDSS will be designed and evaluated in iterations. As this approach to CDSS design for CAMHS is relatively novel, to ensure IDDEAS is usable and appropriate for clinicians and patients, all iterations will be conducted collaboratively among the technical and clinical experts of the IDDEAS Consortium (50). With IDDEAS being an innovation project, each stage will build upon the previous one, with first identifying the needs of practitioners and assessing the perceived usability of the prototype system before going on to investigate the utility and efficacy of the system to care for real patients (50).

Preserving patient confidentiality is a fundamental project requirement. To mitigate the risk of re-identification we will seek to model patient trajectories in a way that reduces the patient representation to a set of care events (e.g., physiological findings and health care system interactions). These will then be clustered so that we operate with representations of similar patient trajectories rather than unique trajectories tied to single individuals.

In developing the project, it was important first to consider the previously encountered challenges of successful CDSS implementations and then evaluate them within the context of the Norwegian local approach to CAMHS. A contribution of this paper is a framework to discuss which considerations a CDSS for local CAMHS must consider both for design and implementation: the involved stakeholders, how they share information, the explicit and implicit “cognitive collaboration” involved and how to address the longitudinal component of patient care. We recognize that some of these challenges, e.g., the handling of comorbidities or supporting multiple distributed stakeholders, are many-faceted and complex and do not often have straightforward solutions. In the IDDEAS project we seek to use this framework as a foundation for a structured engagement with our clinician partners and ultimately better understand the context and processes of CAMHS. We believe this will help us to understand the design and implementation trade-offs we must make but also where a CDSS can realistically have a positive impact on care delivery.

IDDEAS involves multiple stakeholders, including clinicians, researchers, computer engineers, service-user organization representatives, among others, and aims to facilitate “cognitive collaboration” throughout the project. While designated responsibilities lead to differing extents of active involvement from these stakeholders, the IDDEAS Consortium holds regular collaborative meetings for all stakeholders to consistently include multidisciplinary perspectives through development, evaluation and implementation. In addition to multidisciplinary cooperation, IDDEAS is nationally funded by the Norwegian Research Council (i.e., Norges Forskingsrådet) and involves collaboration on a national level (i.e., between different regional CAMH clinics), as well as on an international level, with Consortium members representing Norway, the United States, and several countries of the European Union (50, 51).

Overall, IDDEAS proposes an approach to CDSS design and implementation that not only utilizes the local available resources but also builds off of previously-established challenges and limitations of CDSS uptake and use in other settings, to try to avoid past shortcomings while adapting the approach to meet the local CAMHS.

## Discussion

CDSS implementation in CAMHS has the potential to improve the quality of care and clinical outcomes for patients. The complexity of child and adolescent mental health requires a CDSS design that approaches treatment as a long-term, highly complex process. The optimal approach will encourage collaboration among stakeholders, involving their perspectives and knowledge as part of the foundation for the decision-making processes, while ensuring the patient receives appropriate, individualized care. The proposed IDDEAS in Norway offers helpful means to use innovative technology to improve CAMHS. While IDDEAS is first proposed for Norway, the project intends to test the CDSS within Scandinavia and Europe. A CDSS for child and adolescent mental health, designed and implemented based on established evidence, and using the LEaP approach, can result in improving the quality of services and the health of patients.

## Data Availability Statement

The original contributions presented in the study are included in the article/supplementary material, further inquiries can be directed to the corresponding author/s.

## Author Contributions

TR was responsible for establishing the direction and writing the manuscript. CC also contributed substantially to the planning, writing, revising, and finalizing of the manuscript. TR, ØN, and KK contributed to the development of content on computer decision support systems and computer engineering. NS, BL, RK, LF, OW, and VB all contributed to the development of content related to clinical components. NS provided extensive feedback throughout the entirety of the manuscript's development process. All authors contributed to the article and approved the submitted version.

## Conflict of Interest

The authors declare that the research was conducted in the absence of any commercial or financial relationships that could be construed as a potential conflict of interest.
